# A Study on the Residual Stress of the Co-Based Alloy Plasma Cladding Layer

**DOI:** 10.3390/ma15155143

**Published:** 2022-07-25

**Authors:** Youbin Lai, Xiang Yue, Wenwen Yue

**Affiliations:** 1 Intelligent Manufacturing Key Laboratory of Ministry of Education, Shantou University, Shantou 515063, China; 21wwyue@stu.edu.cn; 2 College of Engineering, Shenyang Agricultural University, Shenyang 110866, China; yuexiang@syau.edu.cn

**Keywords:** plasma cladding, residual stress, Co-based alloy, multi-channel scanning, blind-hole method, finite element simulation

## Abstract

The distribution law of residual stress in multi-channel scanned plasma cladding of Co-based alloy under different process parameters was studied by means of simulation and tests, and the optimum process parameters were optimized. The simulation model of the plasma cladding stress field was established by ABAQUS software, and the influence law of the working current, scanning speed, and scanning mode on the residual stress of the Co-based alloy multi-channel scanning was studied. A set of optimal cladding process parameters were obtained. The residual stress of the cladding layer was measured by the blind hole method and compared with the stress value in the finite element model. The results show that there is residual tensile stress on the surface of the cladding layer. The residual stress along the direction of the scanning path is greater than that along the direction of the scan sequence. The residual stress increases with the increase of the working current. The scanning speed is greater, and the residual stress is smaller. The residual stress of the short-edge scanning is greater than that of the long-edge scanning. The residual stress of the successive scanning is greater than that of the reciprocating scanning. The long-edge reciprocating scanning is the best scanning mode. The best combination of process parameters is the working current of 90 A, the scanning speed of 100 mm/min, and the long-edge reciprocating scanning mode.

## 1. Introduction

Co-based alloy self-fluxing powder has good resistance to wear, corrosion, and high temperature oxidation resistance [1,2]. It is widely used in the aerospace, automotive, and railroad industries [3]. Co-based coating by plasma cladding has advantages that cannot be replaced by other processing technologies, such as a high arc stability, a low thermal deformation, an excellent interfacial bonding ability, and lower equipment and operating costs, which have broad application prospects in surface modification [4]. Plasma cladding technology is an advanced surface modification and repair technology, the principle of which is to use high-energy plasma beams to melt the substrate with its surface metal powder by heat and form a solid metallurgical coating after rapid solidification, thus substantially improving the performance of the substrate [5,6,7,8]. Due to the process characteristics of rapid heating and cooling of the molten pool part in the plasma cladding process, the rapid cooling of the layer and the difference in thermal expansion coefficients between the substrate and the clad layer can cause accumulated residual stresses, and the larger tensile residual stresses induce fatigue cracks, reducing the wear resistance and peeling resistance of the clad layer [9,10], causing deformation and cracking of the substrate [9] and affecting the subsequent mechanical processing of the substrate and its serviceability and life [11]. Therefore, it is of great significance to study and to regulate the distribution law of residual stress in plasma cladding.

At present, there are two main research methods for the residual stress of plasma cladding. One is to adopt the pure experiment method. After measuring the residual stress of the cladding layer, analyze the influence of different process parameters on the residual stress and explore the distribution law of residual stress at each position of the cladding layer. Because of its simple and direct characteristics, this research method is widely used. However, as plasma cladding is a complex thermal processing technology, the stress field in the cladding process changes transiently with time. The experimental measurement method cannot obtain the transient change law of residual stress. Moreover, the number of measurement points on the cladding layer is limited, which cannot fully reflect the distribution of residual stress at each position of the cladding layer. Therefore, this research method has great limitations. Another way to study the residual stress of plasma cladding is to refer to the finite element analysis method of the laser cladding stress field. The finite element simulations can provide information on the variation of residual stresses with process parameters (e.g., working current, scanning speed, scanning path, etc.). The optimum process parameters or their range can be predicted before the experiment. In addition, the finite element simulations provide an effective guide for the experiments, avoiding unnecessary time and material consumption due to the multiple combinations of process parameters for repeated experiments.

Li Y. [12] and others have used FV520B as the substrate and ABAQUS software to establish the simulation model of Co-based alloy single-channel plasma cladding. The stress distribution of the model after a thermal cycle is analyzed, and the thermal fatigue resistance of the cladding layer at different temperatures is explored. Zhang Z. [13] established the finite element model of thermal mechanical coupling laser cladding, and he studied the stress evolution of the Co-based alloy cladding layer. He summarized the stress distribution law of single-channel flat plate laser cladding, double-channel flat plate laser cladding, and double-channel cylindrical surface cladding. It is concluded that preheating can reduce the residual stress of single-channel and double-channel flat plate laser cladding, but it cannot affect the residual stress of double-channel cylindrical cladding. Wang Lifang [14] and others have established a single-channel laser cladding model by ANSYS software. The influence law of laser power, scanning speed, and spot size on the residual stress of the cladding layer is studied by the orthogonal test method, and the process parameters are optimized. Liu Xiaodong [15] established the finite element model of multi-channel scanning laser cladding by ANSYS software, designed different scanning routes for a multi-channel scanning simulation test of laser cladding, optimized the best scanning route, and concluded that the pre-bending substrate can greatly reduce the residual stress of the cladding layer.

In view of the above research status, at present, the research on multi-channel scanning plasma cladding finite element simulation and residual stress is very scarce at home and abroad. Based on this, the research method of combining a finite element simulation and test is taken in this paper to explore the optimal combination of plasma cladding process parameters and the best scanning mode. The method of stress relief annealing is used to regulate the residual stress. Finally, another sample was prepared with the optimal process parameters.

## 2. Establishment of Finite Element Model

### 2.1. Simplification of Finite Element Model

The plasma cladding process is very complex, and the nonlinear change of material and heat transfer can easily lead to a divergence and a distortion of the calculation results. In order to obtain more realistic simulation results, the model established in this paper was simplified, and it was assumed:

The cladding powder was isotropic with the substrate material;
The convective heat transfer coefficient was constant in the cladding simulation process;
The vaporization effect of the cladding powder was not considered;
The whole cladding process followed the Van Mises stress criterion;
The moving heat source was always stable;
The interaction between process parameters was ignored.

### 2.2. Simplification of Finite Element Model

**Figure 1:**
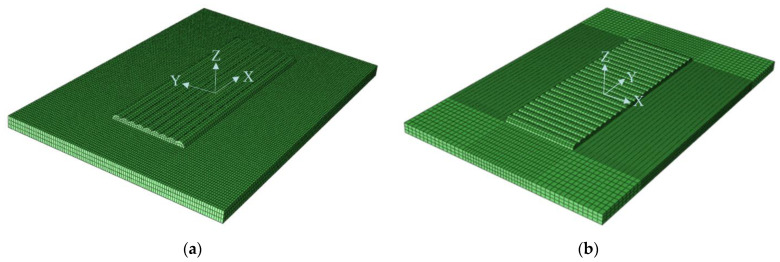
Finite element model: (**a**) Long-edge scanning; (**b**) Short-edge scanning.

The finite element three-dimensional geometric model of multi-channel scanning plasma cladding was established by ABAQUS software (V6.10, Shenyang, China), and it set the substrate material properties to Q235 and the cladding layer to Co-based alloy. Then, the geometric model was meshed by ABAQUS software. The finite element model is shown in Figure 1 in which the size of the substrate is 115 mm × 95 mm × 10 mm. The single-channel cladding layer is 5 mm wide, 2.5 mm high, and 90 mm long. In order to ensure the accuracy and the efficiency of the finite element calculation, the mesh was divided by pure hexahedron. The grid size of the area around the molten pool was 2 mm. The total number of simulation model elements was 41,498, and the total number of nodes was 53,726. The element type was the eight-node hexahedron C3D8R linear shrinkage integral structure analysis element with an hourglass control function. The overall stress state of the substrate and the cladding is calculated by the finite element simulation method.

### 2.3. Imposition of Boundary Conditions

**Figure 2:**
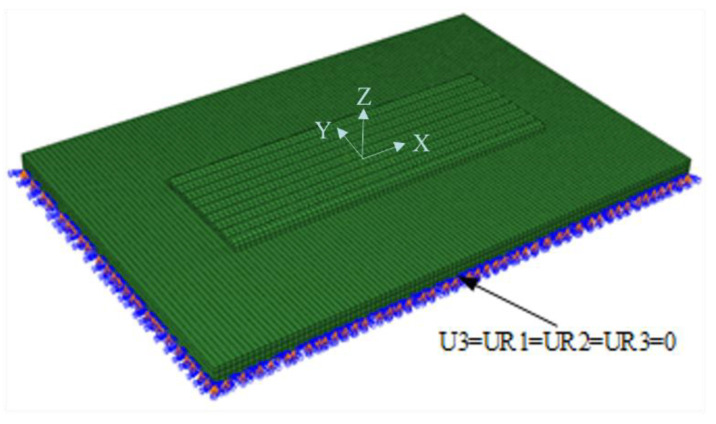
Applying displacement constraints on the substrate.

The boundary conditions of the plasma cladding simulation process mainly include a convective radiation condition in the thermal analysis and a displacement boundary condition in the force analysis. A convective radiation condition means that the influence of the heat exchange between the substrate surface and the surrounding environment on the calculation results is fully considered in the simulation. When the substrate temperature is low, the main heat transfer mode is heat conduction. With the cladding, the substrate is thickening, and the heat transfer is dominated by heat radiation at this time. The ambient temperature in this analysis was 20 °C, and the radiation coefficient was 0.8. In the stress field analysis, the displacement of the substrate was constrained according to the actual situation. As shown in Figure 2, the horizontal displacement of the substrate in the Z direction is constrained, and the rotation constraint is applied to the three directions of X axis, Y axis, and Z axis.

### 2.4. Mobile Heat Source

**Figure 3:**
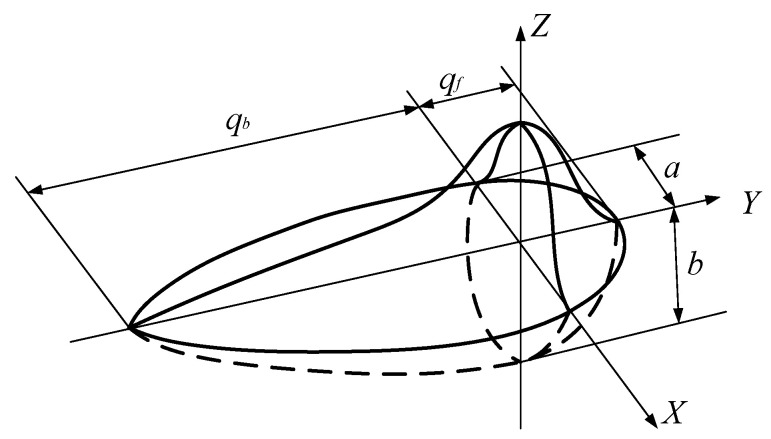
Double ellipsoid heat source.

The heat source model is the core part of the surface cladding simulation. After years of development, it developed from the initial one-dimensional point heat source and line heat source to a two-dimensional surface heat source and a three-dimensional volume heat source. With continuous breakthroughs and innovations in the heat source model, the simulation results are closer to the reality; but, at the same time, it also greatly increases the simulation workload. Therefore, an appropriate mobile heat source model should be selected before the simulation analysis. A double ellipsoidal heat source (see Figure 3) was selected in this paper. Additionally, it mainly takes into account the asymmetric characteristics of the leading and the trailing edges of the melt pool, and it uses two ellipses with different semi-long axes to characterize the energy distribution in the front and the rear halves of the heat source model, which ensures the reliability of temperature and stress calculations.

The double ellipsoid heat source is divided into two sections. The expression of the first half section is:

(1) $$q_{f}\left(x,y,z\right)=\frac{6\sqrt{3}f_{r}q_{0}}{abc_{f}\pi \sqrt{\pi }}\exp\left(-\frac{3x^{2}}{c_{f}^{2}}-\frac{3y^{2}}{a^{2}}-\frac{3z^{2}}{b^{2}}\right)$$

The expression of the latter half is:

(2) $$q_{b}\left(x,y,z\right)=\frac{6\sqrt{3}f_{b}q_{0}}{abc_{b}\pi \sqrt{\pi }}\exp\left(-\frac{3x^{2}}{c_{b}^{2}}-\frac{3y^{2}}{a^{2}}-\frac{3z^{2}}{b^{2}}\right)$$

where $a,b,c_{f},c_{b}$ is the ellipsoidal shape parameter of the heat source; $f_{r},f_{b}$ is the heat ratio coefficient of the ellipsoid before and after the heat source and after $f_{r}+f_{b}=2$. Generally, $f_{r}=0.6$, $f_{b}=1.4$, $q_{0}$ is the heat input power. The expression is:

(3) $$q_{0}=\eta UI$$

where U is the voltage of the plasma stack welding machine, V; I is the working current, A; and η is the thermal efficiency of arc, 0.8 for the plasma cladding stack welding machine, generally.

According to the above formula, the heat flux density of the first half of the heat source is smaller than that of the latter half. The heat flux distribution of the cladding layer can be adjusted by changing the parameters of the ellipsoid, which ultimately determines the morphology of the molten pool. Because ABAQUS software does not have the function of a moving heat source, it is necessary to insert a double ellipsoid heat source model into the DFLUX interface of the software through a third-party software. In this paper, Fortran computer language was used to write the mobile heat source subroutine that conformed to the actual situation, according to the characteristics of the cladding heat source, and the interface with DFLUX was interactive to realize the loading of the mobile heat source of the plasma cladding simulation model.

## 3. Stress Field Analysis of Plasma Cladding

**Figure 4:**
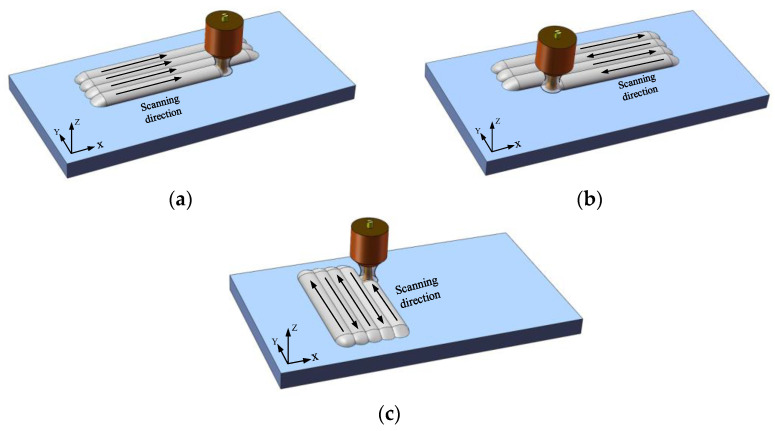
Different scanning modes: (**a**) Successive scanning; (**b**) Long-edge scanning; (**c**) Short-edge reciprocating scanning.

A single factor test was designed to simulate the stress field of plasma cladding. The test scheme is shown in Table 1. Simulation tests for groups No. 1, No. 2, and No. 3 were used to analyze the influence law of the working current on residual stress. Simulation tests for groups No. 2, No. 4, and No. 5 were used to analyze the influence law of the scanning speed on residual stress. Simulation tests for group No. 2, No. 6, and No. 7 were used to analyze the influence law of the scanning mode on residual stress. The three scanning modes are shown in Figure 4.

### 3.1. Influence of Working Current on Residual Stress

**Figure 5:**
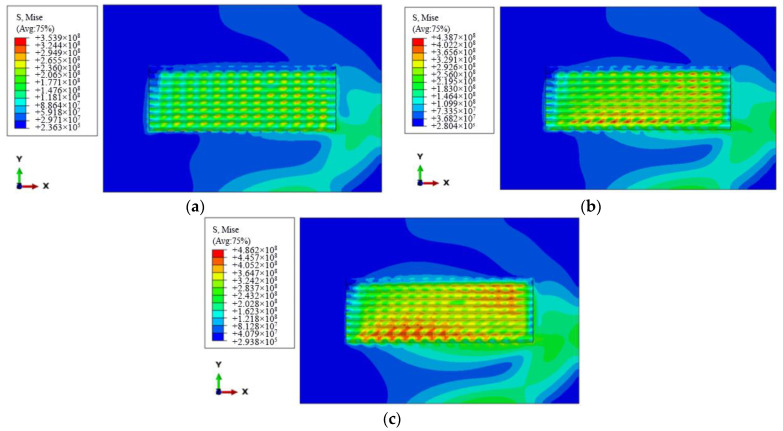
Influence nephogram distribution of working current on stress field: (**a**) working current 90 A; (**b**) working current 95 A; (**c**) working current 100 A.

According to Table 1, the influence of the working current on the residual stress of cladding layer is to be analyzed; the plasma cladding stress field is simulated by long-edge successive scanning, according to the simulation test parameters of groups No. 1, No. 2, and No. 3, that is, the working current is 90 A, 95 A, and 100 A, respectively, and the scanning speed is 90 mm/min. Figure 5a–c is the residual stress nephogram distribution. It can be seen from the figures that when other process parameters are constant, the maximum residual stress of the cladding layer is 354 MPa, 439 MPa, and 486 MPa, respectively, with the increase of the working current, showing a rising trend. This is because the melting degree of the substrate and the power will be directly affected by the work current. The increase in the working current will lead to the powder and the substrate melting more fully, making the width of the melt pool wider. At the same time, the temperature difference between the substrate and the melt pool becomes larger, making the melt pool cooling plastic shrinkage and residual stresses become larger. Therefore, on the premise of ensuring the forming quality, a lower working current should be selected during cladding.

### 3.2. Influence of Scanning Speed on Residual Stress

**Figure 6:**
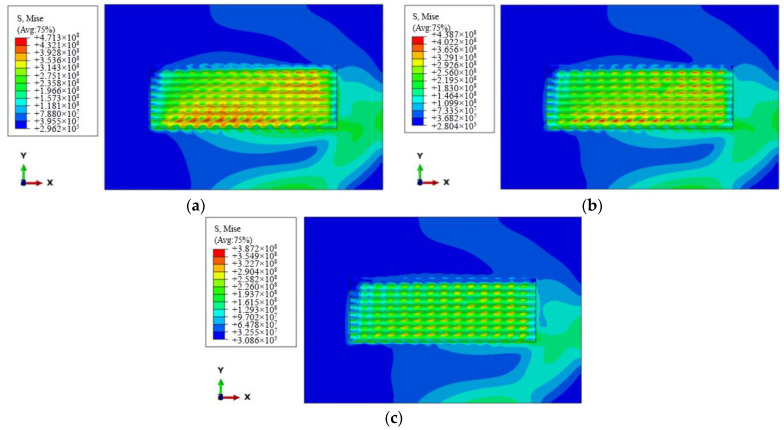
Influence nephogram distribution of scanning speed on stress field: (**a**) scanning speed 80 mm/min; (**b**) scanning speed 90 mm/min; (**c**) scanning speed 100 mm/min.

According to Table 1, the influence of the scanning speed on the stress field of the cladding layer is to be analyzed; the plasma cladding stress field is simulated by long-edge successive scanning, according to the simulation test parameters of groups No. 2, No. 4, and No. 5., that is, the scanning speed is 80 mm/min, 90 mm/min, and 100 mm/min, respectively, and the working current is 95 A. Figure 6a–c is the nephogram distribution of residual stress. It can be seen from the figures that when other process parameters are constant, the maximum residual stress of each group is 471 MPa, 439 MPa, and 387 MPa, respectively, with the increase of scanning speed, showing a downward trend. The reason is that with the increase of the scanning speed, the heat of molten pool per unit time decreases. Increasing the scanning speed under the premise of a constant powder feeding speed will make the width of the molten pool smaller. The above two reasons will reduce the deformation of the molten pool during cooling, resulting in a decrease in the residual stress of the cladding layer. Therefore, on the premise of ensuring the quality of the cladding layer, using a larger scanning speed can effectively reduce the residual stress.

### 3.3. Influence of Scanning mode on Residual Stress

**Figure 7:**
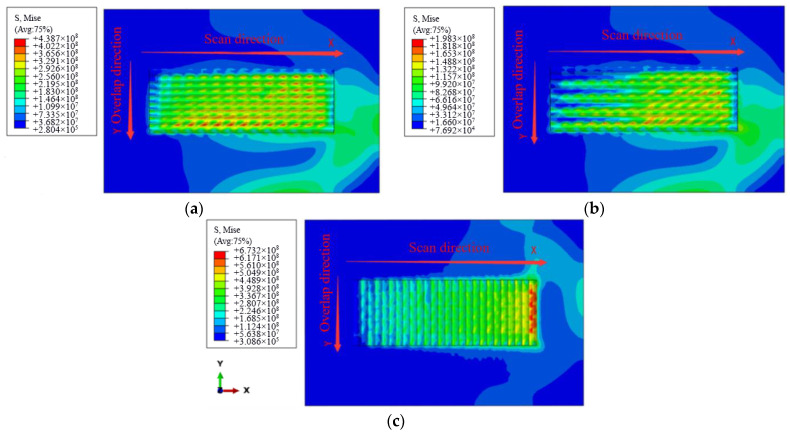
Residual stress nephogram distribution: (**a**) Long-edge successive scanning; (**b**) Long-edge reciprocating scanning; (**c**) Short-edge reciprocating scanning.

Figure 7 shows the residual stress distribution of the cladding layer under three scanning modes. Comparing Figure 7a with Figure 7b, it can be seen that the maximum residual stress of the cladding layer is 198 MPa obtained by the reciprocating scanning mode. It is much smaller than the maximum residual stress of 439 MPa obtained by the successive scanning. Especially in the scanning area of two cladding layers, the temperature difference in this area is low, and the thermoplastic deformation of the molten pool is small when reciprocating scanning mode is adopted. Therefore, the residual stress generated is small when the whole cladding layer reaches the stress balance again. The residual stress at the beginning and at the end of the cladding layer in Figure 7b is very small. This distribution state can effectively prevent the warpage of the cladding layer and make it more firmly combined with the substrate. By observing Figure 7c, it can be seen that the maximum residual tensile stress of the cladding layer by short-edge scanning is up to 673 MPa, which is much higher than that by long-edge scanning. Especially in the last cladding layer, there is a large collection of residual stress, which greatly reduces the firmness of the combination between the cladding layer and the substrate, and the cladding layer is prone to warping and falling off. In conclusion, long-edge reciprocating scanning is the best scanning mode.

## 4. Verification Test

### 4.1. Test Materials

**Figure 8:**
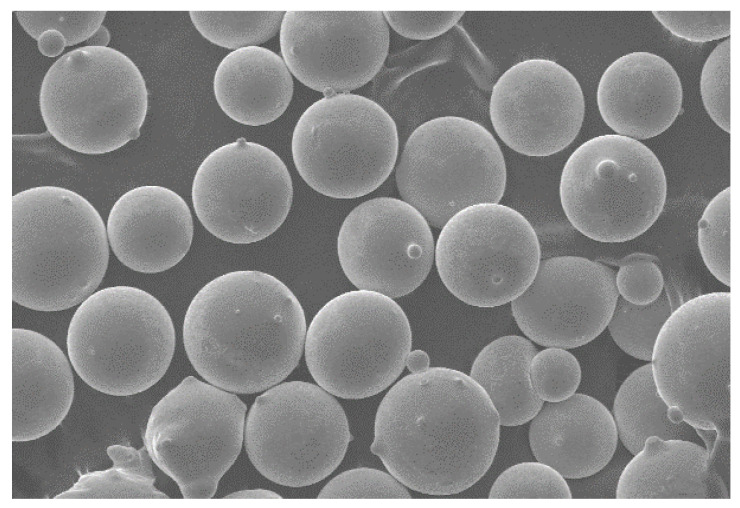
SEM morphology of Co-based alloy powder (220).

The substrate for the verification test was made of Q235 steel with a size of 150 mm × 75 mm × 8 mm. Before the test, the substrate was subjected to stress relief annealing to eliminate the original residual stress in it. The substrate was ground and polished to remove the surface oxide layer and to increase its surface finish after heat treatment. Then, it was further cleaned with acetone [16]. The Co-based alloy powder particles used for cladding were spherical (as shown in Figure 8), with a particle size of 100–270 mesh and a hardness of 40–44 HRC. The composition (in mass fraction) is shown in Table 2.

### 4.2. Sample Preparation

**Figure 9:**
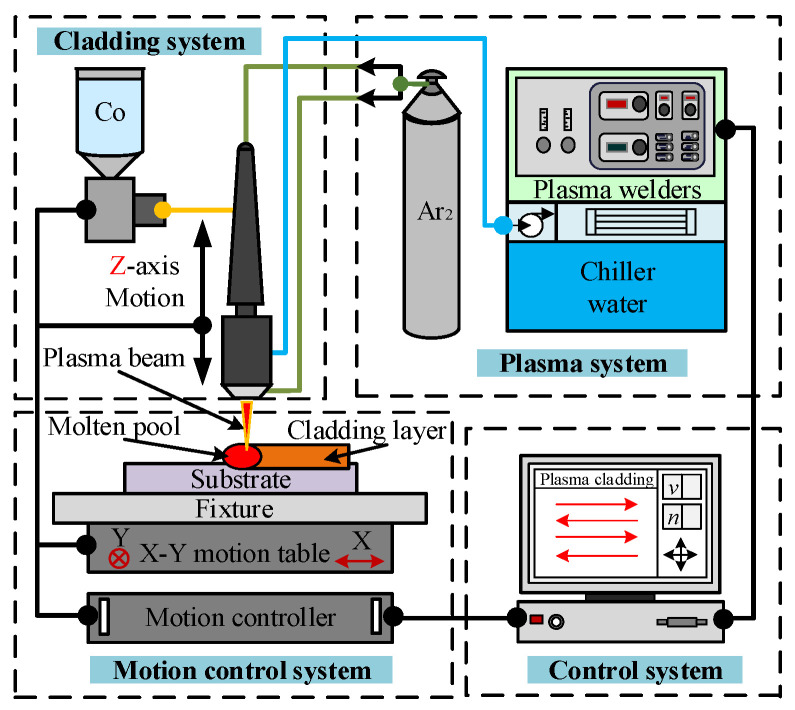
Schematic diagram of plasma cladding system.

The cladding test was carried out on the plasma cladding system of Shenyang Agricultural University (as shown in Figure 9). In order to remove the moisture in the powder and to enhance the fluidity and the uniformity of the powder during transmission, the powder was screened twice before the test and dried under a 120 °C vacuum environment [17]. The influence of the working current, scanning speed, and scanning mode on residual stress was studied by a single factor test. Other process parameters in the test were as follows: ion gas flow was 0.7 L/h, powder feeding gas flow was 3 L/h, protective gas flow was 5 L/h, the nozzle was 50 mm away from the specimen, and the scanning rate was 35%.

Seven groups of plasma cladding specimens (as shown in Table 3) were prepared by a single factor test. For the combination of groups No. 1, No. 2, and No. 3, process parameters were used to analyze the influence of the working current on the residual stress. For the combination of groups No. 2, No. 4, and No. 5, process parameters were used to analyze the influence of the scanning speed on the residual stress. For the combination of groups No. 2, No. 6, and No. 7, process parameters were used to analyze the influence of the scanning mode on the residual stress.

### 4.3. Residual Stress Measurement by Blind Hole Method

**Figure 10:**
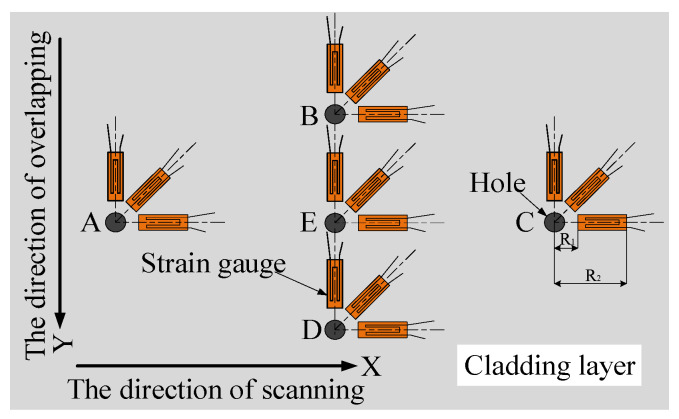
Schematic diagram of residual stress test.

**Figure 11:**
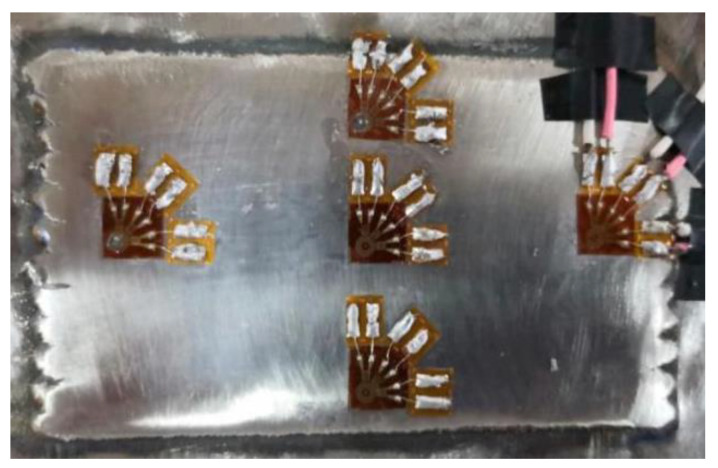
Sample of residual stress test.

The residual stress at different positions of the cladding layer was measured by the blind hole method, using a BX120-1CG resistance strain gauge and a JYB-1 digital static strain gauge. The test process was as follows:

(1) The strain gauge was pasted at the position where the residual stress of the cladding layer was to be measured. Before the strain gauge was pasted, the surface of the cladding layer was polished, and acetone was used for cleaning the cladding layer to ensure that the strain gauge could be closely adhered to it. After the strain gauge was pasted with 502 glue, it would be placed for 24 h before subsequent operation. (The strain gauge placement position is shown in Figure 10).

(2) The indicator of the strain gauge was adjusted to zero, and the borehole was started. In order to reduce the influence of the plastic deformation at the borehole edge on the measurement accuracy [18], the distance between the borehole center and the sensitive grating of the strain gauge was 2 mm. Firstly, a drill bit with a diameter of 1 mm was selected to determine the position of the hole center, and then the drill bit with a diameter of 2 mm was used for drilling. The drilling depth was 2 mm. (The residual stress test sample is shown in Figure 11).

(3) The additional strain generated by drilling heat during drilling would have a certain impact on the reading, so it should be kept for 20 min after drilling. After the hole edge returned to the initial temperature, a group of readings was read every 5 min until the adjacent two readings were the same. The average value of the three groups of values was taken as the strain value [19].

### 4.4. Calculation of Residual Stress

The main calculation formula of residual stress is as follows [20]:

(4) $$\sigma_{1,2}=E\left[\frac{\varepsilon_{1}+\varepsilon_{3}}{4A}\mp \frac{1}{4B}\sqrt{{\left(\varepsilon_{1}-\varepsilon_{3}\right)}^{2}+{\left(2\varepsilon_{2}-\varepsilon_{1}-\varepsilon_{3}\right)}^{2}}\right]$$

(5) $$\theta =\frac{1}{2}\arctan\frac{2\varepsilon_{2}-\varepsilon_{1}-\varepsilon_{3}}{\varepsilon_{3}-\varepsilon_{1}}$$

(6) $$\sigma_{x}=\frac{\sigma_{1}+\sigma_{2}}{2}+\frac{\sigma_{1}-\sigma_{2}}{2}\cdot \cos2\theta$$

(7) $$\sigma_{y}=\frac{\sigma_{1}+\sigma_{2}}{2}-\frac{\sigma_{1}-\sigma_{2}}{2}\cdot \cos2\theta$$

where $\varepsilon_{1}$, $\varepsilon_{2}$, $\varepsilon_{3}$ is the strain released in the three directions of the strain gauge, respectively; $\sigma_{1}$, $\sigma_{2}$ is the maximum principal stress and the minimum principal stress, respectively; and $\sigma_{x}$, $\sigma_{y}$ is the X-direction stress and the Y-direction stress, respectively. A, B is the stress release coefficient, and its calculation formula is shown in Equations (8) and (9).

(8) $$A=-k\frac{1+v}{2}\frac{a^{2}}{r_{1}r_{2}}$$

(9) $$B=-\frac{2a^{2}}{r_{1}r_{2}}\left[-1+\frac{1+v}{4}\frac{a^{2}\left(r_{1}^{2}+r_{1}r_{2}+r_{2}^{2}\right)}{r_{1}^{2}r_{2}^{2}}\right]$$

where *E* is the elastic modulus of the cladding material (207.65 GPa); *v* is Poisson’s ratio of cladding material (0.25); *a* is the drilling radius (1 mm); and $r_{1}$, $r_{2}$ is the nearest and the furthest distance from the strain gauge sensitive gate to the hole center ($r_{1}$ = 2 mm, $r_{2}$ = 3 mm).

The residual stress is calculated according to the above formula, and the results are shown in Table 4.

It can be seen from Table 4 that the residual stress on the surface of the cladding layer is tensile stress, and the residual tensile stress along the scanning path direction is greater than that along the scanning direction. In the plasma cladding process, the molten pool and its surrounding area generate a certain plastic deformation after thermal expansion under the action of a high-energy plasma arc. The surrounding substrate restricts the solidification shrinkage of the molten pool during cooling, resulting in residual tensile stress in the cladding layer. The experimental results are consistent with the simulation analysis, and the simulation model fits well.

### 4.5. Influence of Working Current on Residual Stress

**Figure 12:**
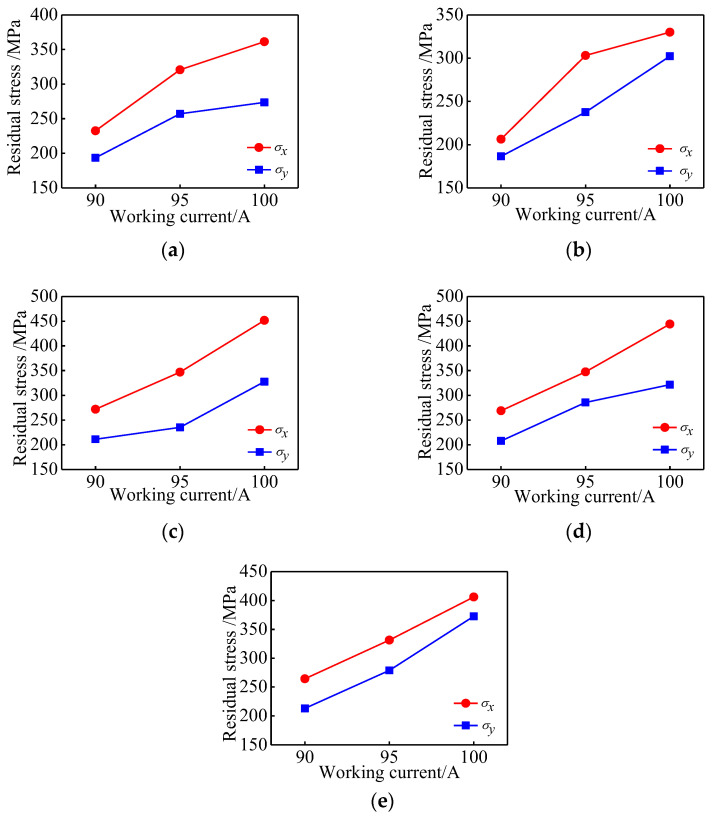
Residual stress at different working currents: (**a**) Test point A; (**b**) Test point B; (**c**) Test point C; (**d**) Test point D; (**e**) Test point E.

Figure 12 shows the change in the residual stress on the surface of the sample by plasma cladding with a 90 A, 95 A, and 100 A working current, respectively, when the scanning speed is 90 mm/min and the long edge is scanned step by step. It can be seen that the residual stresses in the X and the Y directions at each position of the test sample increase with the increase of the working current. The law obtained from the verification test fits well with the simulation results.

### 4.6. Influence of Scanning Speed on Residual Stress

**Figure 13:**
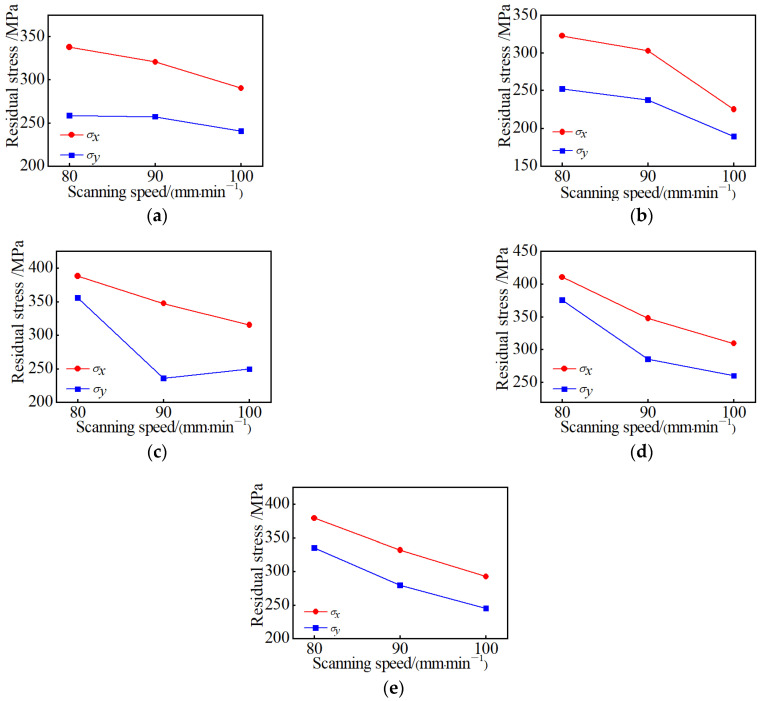
Residual stress at different scanning speeds: (**a**) Test point A; (**b**) Test point B; (**c**) Test point C; (**d**) Test point D; (**e**) Test point E.

Figure 13 shows the change of the residual stress on the surface of the sample by plasma cladding at the scanning speed of 80 mm/min, 90 mm/min, and 100 mm/min, respectively, when the working current is 95 A and the long edge is scanned step by step. It can be seen that with the increase in the scanning speed, the residual stress in the X and the Y directions at each position of the test sample shows a downward trend. It is consistent with the simulation results. Therefore, a larger scanning speed can effectively reduce the residual stress.

### 4.7. Influence of Scanning mode on Residual Stress

**Figure 14:**
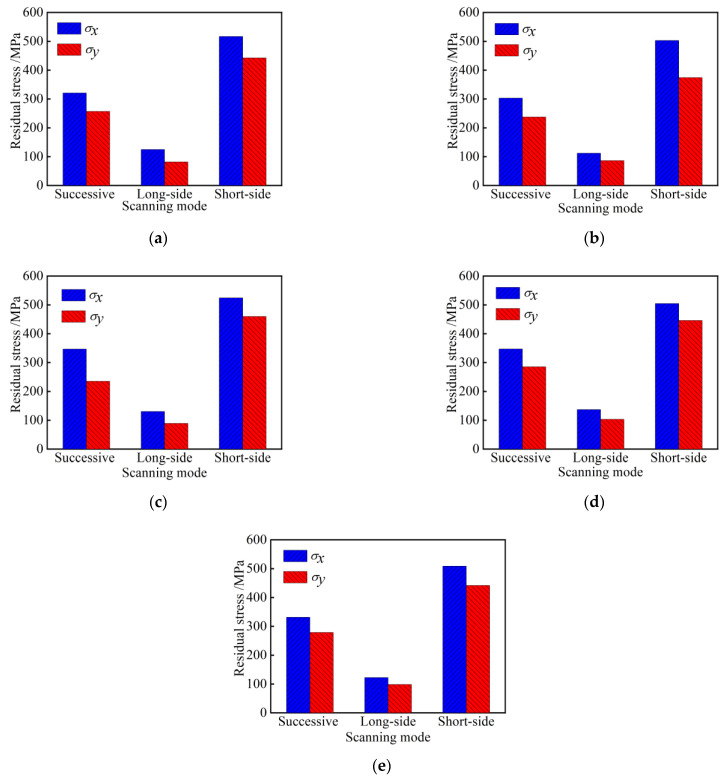
Residual stress at different scanning modes (**a**) Test point A; (**b**) Test point B; (**c**) Test point C; (**d**) Test point D; (**e**) Test point E.

Figure 14 shows the change of the residual stress on the surface of the sample by plasma cladding, when the working current is 95 A and the scanning speed is 100 mm/min. The plasma cladding samples are carried out by long-edge scanning, long-edge reciprocating scanning, and short-edge reciprocating scanning, respectively. It can be seen from the figures that the residual stress under the successive scanning mode is greater than that under the reciprocating scanning mode. The residual stress of short-edge scanning is greater than that of long-edge scanning. The law is consistent with the simulation results. Therefore, long-edge reciprocating scanning is the best scanning mode.

**Figure 15:**
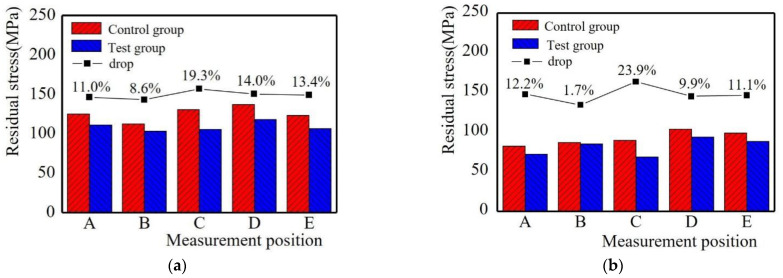
Verification test: (**a**) Residual stress in X direction; (**b**) Residual stress in Y direction.

From the above analysis, it can be predicted that the residual stress of the cladding layer is the smallest under the combination of process parameters of working current 90 A, scanning speed 100 mm/min, and long-edge reciprocating scanning mode. The cladding test is carried out under this working condition. The residual stress of the sample cladding layer is measured and compared with the group with the smallest residual stress mentioned above (working current 95 A, scanning speed 100 mm/min, long-edge reciprocating scanning) (the results are shown in Figure 15). The rationalization of the process parameters can significantly modulate the residual stresses. At the same time, the working current influences the central temperature of the melt pool and the cooling rate, thus causing differences in the temperature gradient. A larger temperature gradient leads to an increase in plastic shrinkage and residual stresses in the melt pool. After comparison, the residual stress of the sample in the prediction group is smaller than that in the controlled group, which proves that the prediction is correct.

## 5. Conclusions

(1) The residual stress on the surface of the cladding layer is tensile stress, and the residual stress along the scanning path is greater than that along the scanning direction ($\sigma_{x}$ > $\sigma_{y}$). Both directions are the direction of increasing residual stress.
(2) The residual stress increases with the increase of the working current. The scanning speed is larger, and the residual stress is smaller. The residual stress of the short-edge scanning mode is greater than that of the long-edge scanning mode. The residual stress in a successive scanning mode is greater than that in a reciprocating scanning mode. The residual stress of the specimen obtained by long-edge reciprocating scanning is the smallest, which is the best scanning mode.
(3) The working current 90 A and the scanning speed 100 mm/min is the best combination of process parameters. Long-edge reciprocating is the best scanning mode.

## Figures and Tables

**Table 1:** Design of plasma cladding simulation test.

No.	Working Current/A	Scanning Speed/(mm·min^−1^)	Scanning Mode	Scanning Rate/%
1	90	90	Successive	35
2	95	90	Successive	35
3	100	90	Successive	35
4	95	80	Successive	35
5	95	100	Successive	35
6	95	90	Long-side	35
7	95	90	Short-side	35

**Table 2:** Chemical composition of Co-based alloy powder (mass fraction/%).

C	Cr	Fe	Mn	Mo	Ni	Si	W	Co
1.11	28.61	0.45	0.24	0.21	2.55	1.41	4.67	Bal

**Table 3:** Process parameters of the plasma cladding.

No.	Working Current /A	Scanning Speed /(mm·min^−1^)	Scanning Mode	Test Samples
1	90	90	Successive	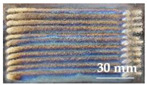
2	95	90	Successive	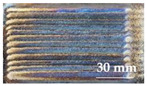
3	100	90	Successive	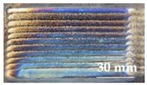
4	95	80	Successive	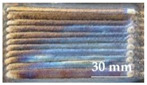
5	95	100	Successive	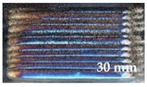
6	95	90	Long-edge	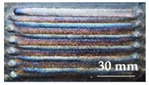
7	95	90	Short-edge	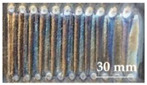

**Table 4:** Presumed value of residual stress.

No.	Simulated Stress Values	A		B		C		D		E	
	*σ* /MPa	*σ_x_* /MPa	*σ_y_* /MPa	*σ_x_* /MPa	*σ_y_* /MPa	*σ_x_* /MPa	*σ_y_* /MPa	*σ_x_* /MPa	*σ_y_* /MPa	*σ_x_* /MPa	*σ_y_* /MPa
1	354	233	194	206	186	272	211	269	208	26	213
2	439	321	257	303	238	347	235	348	286	331	279
4	486	338	258	323	252	388	355	411	376	379	335
5	471	290	241	225	190	315	249	310	260	292	245
6	387	125	82	112	86	130	89	137	104	123	99
7	673	517	443	506	374	525	460	505	446	509	442

## Data Availability

The data used to support the findings of this study are included within the article.
